# Brachial Artery Endothelial Responses during Early Recovery from an Exercise Bout in Patients with Coronary Artery Disease

**DOI:** 10.1155/2014/591918

**Published:** 2014-03-03

**Authors:** Katharine D. Currie, Robert S. McKelvie, Maureen J. MacDonald

**Affiliations:** ^1^Department of Kinesiology, McMaster University, 1280 Main Street West, Hamilton, ON, Canada L8S 4K1; ^2^Department of Medicine, McMaster University, 1280 Main Street West, Hamilton, ON, Canada L8S 4K1; ^3^Hamilton Health Sciences, 237 Barton Street East, Hamilton, ON, Canada L8L 2X2

## Abstract

This study examined the acute endothelial responses to an exercise bout in coronary artery disease (CAD) patients. Nineteen males with CAD (63 ± 8 years) were assessed at rest and 15 minutes following a submaximal exercise bout (recovery). Brachial artery endothelial-dependent function was assessed using flow-mediated dilation (FMD). Brachial artery diameters and velocities were measured using Duplex ultrasound at baseline, and for 3 minutes following a 5-minute ischemic period. Endothelial-independent function was assessed using a 0.4 mg dose of nitroglycerin (NTG). FMD responses were unchanged from rest to recovery; however, there were 2 types of responses: negative and positive FMD responders. Post-hoc analysis revealed that positive responders had lower resting FMD compared to negative responders (3.2 ± 1.7 versus 6.0 ± 2.5%, *P* < 0.05). NTG-mediated dilation was reduced in recovery (22.0 ± 5.6 versus 14.4 ± 5.7%, *P* < 0.001 for rest versus recovery). In conclusion, acute endothelial-dependent responses to submaximal exercise are affected by the degree of resting endothelial dysfunction. The observation of attenuated NTG-mediated dilation during recovery is novel and warrants the investigation of possible mechanisms and clinical significance. Furthermore, it highlights the necessity of both endothelial-dependent and endothelial-independent assessments when evaluating endothelial function changes with an intervention.

## 1. Introduction

Patients with coronary artery disease (CAD) are characterized as having endothelial dysfunction, which describes a decreased capacity of the endothelium to elicit vasodilation [1]. The degree of endothelial dysfunction can be assessed noninvasively using a combination of techniques. Specifically, brachial artery flow-mediated dilation (FMD) provides a measure of endothelial-dependent function which has been shown to be largely mediated by the potent vasodilator nitric oxide (NO) [2] and is an accepted surrogate for coronary artery endothelial-dependent function [3]. Conversely, endothelial-independent function can be measured using an exogenous NO donor such as nitroglycerin (NTG) and is commonly assessed in combination with FMD as a control test to determine whether changes in vasodilation are attributed to the endothelial or vascular smooth muscle layers [4]. FMD has been shown to be attenuated in patients with CAD relative to healthy individuals [2, 5–7], while NTG-mediated dilation has been shown to be attenuated [5, 7, 8] or maintained [2, 6].

In patients with CAD, an attenuated FMD response is associated with an increased risk for future cardiovascular events and death [9–11]. While there is definitive evidence that chronic exercise training improves FMD in CAD patients [12–15], the FMD responses to a single bout of exercise in this population are poorly understood. Previous research in non-CAD populations has demonstrated both reductions [16–22] and no change [17, 19, 20, 22] in FMD responses during early recovery from a single bout of exercise. Postexercise reductions in FMD may indicate a period of elevated cardiovascular risk and therefore are worthy of examination in at-risk populations such as patients with CAD. Therefore, the purpose of this study was to examine the brachial artery endothelial-dependent and endothelial-independent responses during the early recovery period (i.e., 15 minutes) following a single bout of submaximal exercise. Based on previous research in clinical populations [18, 19], we hypothesized that FMD would be transiently impaired following the acute bout of exercise, whereas NTG responses would be unchanged.

## 2. Materials and Methods

### 2.1. Participants

Nineteen males with documented CAD were recruited from the Cardiac Health and Rehabilitation Centre at the Hamilton Health Sciences General Site (Ontario, Canada). All patients were recently diagnosed as having CAD, which was defined as having at least one of the following: angiographically documented stenosis ≥50% in at least one major coronary artery; prior history of documented myocardial infarction (MI), percutaneous coronary intervention (PCI), or coronary artery bypass graft (CAGB) surgery; and positive exercise stress test determined by a positive nuclear scan, or symptoms of chest discomfort accompanied by electrocardiographic (ECG) changes of >1 mm horizontal or down sloping ST segment depression. Exclusion criteria included smoking within three months, noncardiac surgical procedure within two months, MI or CABG within two months, PCI within one month, New York Heart Association class II-IV symptoms of heart failure, documented valve stenosis, documented severe chronic obstructive pulmonary disease, symptomatic peripheral arterial disease, unstable angina, uncontrolled hypertension, uncontrolled atrial arrhythmia or ventricular dysrhythmia, insulin requiring diabetes mellitus, and any musculoskeletal abnormality that would limit exercise participation. The study protocol was reviewed and approved by the Hamilton Health Sciences/Faculty of Health Sciences Research Ethics Board, conforming to the Helsinki Declaration on the use of human subjects, and written informed consent was obtained from patients prior to participation.

### 2.2. Study Design

Patients attended 2 testing sessions, separated by 10 ± 6 days. The first visit involved a medically supervised exercise test to determine their peak exercise capacity. The second visit took place between the hours of 0800–1200 and involved the submaximal exercise bout and brachial artery endothelial function assessments. Prior to testing sessions, participants were instructed to fast for at least 8 hours and to abstain from exercise for 24 hours, caffeine and alcohol consumption for 12 hours, and to avoid taking NTG the morning of the visit. All testing was performed in a temperature-controlled room (23.3 ± 1.1°C).

### 2.3. Acute Submaximal Exercise Bout

Peak exercise capacity was assessed using a medically supervised graded exercise test to fatigue on a cycle ergometer (Ergoline, Bitz, Germany). Following a brief unloaded warmup, patients cycled at 70 rpm at a workload of 100 kpm for 1 minute, after which workload was increased by 100 kpm every minute until volitional fatigue. Expired gas was analyzed using a semiautomated metabolic cart (*V*max⁡ 229; SensorMedics Corporation, Yorba Linda, CA, USA), and oxygen uptake was determined at peak (VO_2_ peak) from 20-second averages. Heart rate was monitored throughout the test using a 12-lead ECG (MAC 5500; General Electric, Freiburg, Germany).

The exercise intensities for the submaximal exercise bout were based on the peak power output achieved during the medically supervised graded exercise test. Following a 3-minute unloaded warmup on a cycle ergometer (Excalibur Sport V2.0; Lode BV, Groningen, The Netherlands), patients performed 4, 3 minutes stages at increasing intensities of 20%, 40%, 60%, and 80% of peak power output.

### 2.4. Brachial Artery Assessments

All rest and recovery (following the submaximal exercise bout) vascular assessments were performed in the supine position. Resting measurements were performed following 30 minutes of quiet rest, while recovery measurements were initiated 15 minutes following the cessation of submaximal exercise. Heart rate and brachial artery blood pressure were measured throughout testing using a single-lead (CC_5_) ECG (model ML 123; ADInstruments Inc., Colorado Springs, CO, USA) and noninvasive hemodynamic monitor (Nexfin; BMEYE, Amsterdam, The Netherlands).

Brachial artery endothelial-dependent function was assessed using the FMD test, based on previously established guidelines [4, 23]. Duplex ultrasound (Vivid Q; GE Medical Systems, Horten, Norway) was used to capture simultaneous images of the right brachial artery (13 MHz) and blood velocity measurements (4 MHz) throughout the FMD protocol. Preocclusion images of the right brachial artery were collected 3–5 cm proximal to the antecubial fossa for 30 seconds at a frame rate of 7.7 frames·s^−1^. A 5-minute period of ischemia was initiated by inflating a pneumatic cuff positioned on the forearm distal to the antecubial fossa to an occlusion pressure of 200 mm Hg using a rapid cuff inflator (model E20 and AG101; Hokanson, Bellevue, WA, USA). Upon cuff release, duplex postocclusion images and blood velocity measurements were collected continuously for 3 minutes. Duplex images were stored in Digital Imaging and Communications in Medicine (DICOM) format for offline analysis. End-diastolic frames, determined by the R-spike of the ECG trace, were extracted and stacked in a new DICOM file using commercially available software (Sante DICOM Editor, Version 3.0.12; Santesoft, Athens, Greece) and analyzed using semiautomated edge-tracking software (AMS (Artery Measurement System) Image and Data Analysis; Gothenburg, Sweden) as previously described [24]. Preocclusion diameters were determined from the 30-second average. Postocclusion diameters were averaged in rolling 5-cycle bins [25], and peak postocclusion diameter was defined as the maximum 5-cycle average. Absolute and relative FMD were calculated as previously described [24].

Continuous intensity weighted mean blood velocity (MBV) signals were obtained using an external spectral analysis system (model Neurovision 500 M TCD; Multigon Industries, Yonkers, NY, USA) and sampled at 100 kHz using commercially available hardware (Powerlab model ML795; ADInstruments, Colorado Springs, CO, USA). MBV was analyzed offline using LabChart 7 Pro for Windows (Powerlab ML 795; ADInstruments, Colorado Springs, CO, USA) by correcting for the angle of insonation (all ≤ 70°). Mean blood flow was calculated by multiplying brachial artery cross-sectional area by MBV. Baseline blood flow is the average over 30 seconds, while peak blood flow is reported as the maximum 5-cycle average during the 3-minute postocclusion period. Shear rate for each bin was calculated by multiplying the MBV bin by 8 and dividing it by the corresponding reactive hyperemic 5-cycle bin. The reactive hyperemic stimulus until peak dilation was quantified as shear rate area under the curve (AUC).

Endothelial-independent function was assessed through administration of 0.4 mg sublingual spray of NTG applied 10 minutes after cuff release [4]. Longitudinal B-mode images (8 MHz) of the right brachial artery were collected at a frame rate of 22.9 frames·s^−1^ prior to administration of NTG (pre-NTG; 10 cardiac cycles) and for 10 cardiac cycles every minute post-NTG up to 10 minutes. End-diastolic frames were extracted and stacked, and peak-NTG diameters were determined from the average of 10 cardiac cycles at each minute. Absolute and relative NTG-mediated dilation was calculated as previously described [12]. We previously reported day-to-day intraclass correlations coefficients of 0.89 and 0.90 for absolute and relative FMD and NTG-mediated dilation, respectively [24].

### 2.5. Statistical Analysis

Statistical analyses were performed using Statistical Package for Social Science software (version 20.0; IBM Corporation, Armonk, NY, USA). Rest and recovery measures were compared using paired tests. Analysis revealed an increase in brachial artery diameters following the submaximal exercise bout, which may influence the degree of both FMD and NTG-mediated dilation. Subsequently, FMD and NTG-mediated dilation was also analyzed using an ANCOVA, as previously described [26]. Briefly, we logarithmically transformed pre- and peak diameters and calculated the change in diameter on the logged scale. This value was entered as the dependent variable in the ANCOVA with time (rest/recovery) as the fixed value and logarithmically transformed prediameter as a covariate. Covariate adjusted means for diameter change during the FMD and NTG assessments were obtained, back-transformed, and then converted to a “corrected” adjusted percentage change by subtracting 1 from the back-transformed value and multiplying it by 100. Data are presented as mean ± SD, with *P* < 0.05 considered statistically significant.

## 3. Results

Patient characteristics, CAD history, and medications are presented in Table 1. Peak heart rates achieved during their medically supervised graded exercise test represented 79% of their age-predicted heart rate maximum. During the submaximal exercise test, mean heart rate was 95 ± 15 bpm while peak heart rate was 122 ± 23 bpm, which is equivalent to 54 ± 22% and 78 ± 16% of their age-predicted heart rate maximums, respectively.

**Figure 1:**
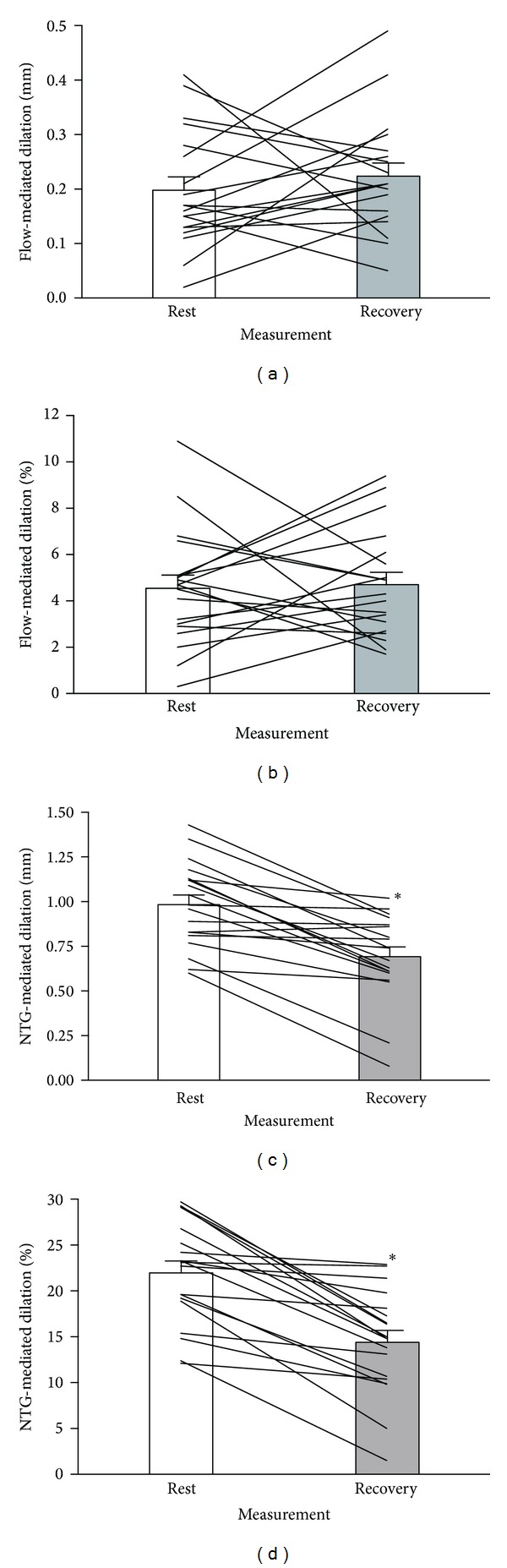
Absolute (mm) and relative (%) brachial artery FMD (a, b) and NTG (c, d) responses at rest (white) and 15 minutes into exercise recovery (grey). Lines represent individual patient responses. *P < 0.001 versus rest.

Endothelial function parameters are presented in Table 2. All brachial artery diameters during the FMD and NTG assessments were increased during recovery compared to rest. However, there was no difference between the FMD preocclusion and pre-NTG diameters at both rest and recovery time points, suggesting that the artery had returned to a “baseline state” prior to administration of NTG. Absolute and relative FMD and NTG responses are presented in Figure 1. FMD was unchanged from rest to recovery, while NTG-mediated dilation was attenuated during recovery compared to rest. “Corrected” FMD and NTG-mediated dilation values are presented in Table 2 and are in agreement with the results in Figure 1. Even when adjusting for preocclusion or pre-NTG diameters, “corrected” FMD was unchanged following submaximal exercise while “corrected” NTG-mediated dilation was decreased. All remaining FMD and NTG indices were unchanged with submaximal exercise. Heart rate and blood pressure indices are also presented in Table 2. Heart rate was elevated during recovery, while systolic and mean arterial pressures were lower. Diastolic blood pressure was unchanged following the submaximal exercise bout.

FMD analysis revealed two distinct groups: individuals who decreased FMD during recovery (negative responders) and individuals who increased FMD during recovery (positive responders). Post-hoc independent *t*-tests revealed no significant differences in patient characteristics or hemodynamics between the two groups; however, all FMD indices (absolute, relative and “corrected” FMD) at rest were lower in the positive responder group compared to the negative responder group (Table 3).

## 4. Discussion

This study provides the first examination of the endothelial responses during early recovery from an exercise bout in patients with CAD. While our FMD findings suggest that endothelial-dependent function is unchanged during the early stages of recovery from exercise, the observation of two distinct types of acute responses suggest that the baseline state of the endothelium may influence the acute response to a bout of exercise. Furthermore, our novel finding of attenuated NTG responses highlights an important mechanistic consideration that has been commonly overlooked by previous investigations.

To date, only 3 studies have examined the time course of brachial artery endothelial responses following exercise. Two of these investigations used an upper limb cuff during the FMD assessment and did not measure NTG-mediated dilation, which limits the ability to draw comparisons [27, 28]. The remaining study examined FMD and NTG responses at 1, 24, and 48 hours after exercise and demonstrated that the reduction in FMD observed at 1 hour after exercise returned to baseline values by 24 hours after exercise and remained stable [29]. While there is obvious merit to examining the responses during an extended period of time after exercise, there is very little information about the time course of endothelial responses during early recovery from exercise. The aim of the present study was to further describe the acute brachial artery endothelial responses to exercise in CAD patients by examining an earlier time point during recovery.

Contrary to our hypothesis, FMD was unchanged during early recovery from exercise, even after adjusting for the postexercise increase in brachial artery diameter. Unchanged FMD values immediately following an exercise bout have been previously reported [17, 19, 20, 22]. The absence of a change in our study is likely attributed to the large variability in individual patient responses (Figures 1(a) and 1(b)). Out of a total of 19 patients, 9 decreased FMD during early recovery from exercise, while 10 increased FMD. Conversely, all 19 patients experience reductions in NTG-mediated dilation during recovery. A comparison between negative and positive FMD responders revealed no significant difference in patient characteristics or resting hemodynamics. Specifically, positive and negative FMD responders were a similar age, exercised at a similar percentage of their age-predicted heart rate maximum during the acute exercise bout, and had similar BMI, fitness, and heart rate and blood pressure values. However, there was a difference in resting FMD such that individuals who had a lower FMD value at rest experienced a postexercise increase in FMD, while individuals who had higher FMD values at rest experienced a reduction in FMD during recovery. This is the first study to demonstrate that the direction change in FMD in response to an acute bout of exercise is dependent on the degree of baseline endothelial dysfunction. However, a previous study demonstrated that cardiac patients with endothelial dysfunction experienced positive responses to an intervention relative to the nonresponses of the sample of cardiac patients with normal endothelial function [30]. Based on our novel observation of positive and negative FMD responders, we believe that additional research is required to delineate what factors influence acute endothelial-dependent responses to a bout of exercise.

To date, there are few reports of increased FMD during early recovery from an exercise bout, and the current results present data at a time point earlier than previous reports. Although it is unknown what mechanisms may mediate this response in our positive responders cohort, increased NO bioavailability [29] and elevated FMD have been observed 60 minutes following an acute bout of exercise in both healthy [31, 32] and CAD [24] individuals. NO bioavailability has been shown to be impaired in individuals with atherosclerosis [33]; therefore, an increase in NO bioavailability immediately following exercise may explain the observed increase in FMD in our positive responders cohort with pronounced endothelial dysfunction. Conversely, several studies have documented postexercise reductions in FMD in both healthy [16–22] and clinical [18–22] populations. Exercise has been shown to increase circulation of reactive oxygen species, including superoxide anions [34, 35], which are capable of scavenging available NO, thereby decreasing its bioavailability. The postexercise reduction in FMD observed in our negative responders cohort, therefore, may be attributed to increases in oxidative stress and decreased NO bioavailability [34–36]. There is also evidence that the FMD test may not be exclusively mediated by NO; therefore, additional factors should be considered as altered sympathetic output may lead to augmented or attenuated FMD responses [37].

Evidence on endothelial-independent function in CAD patients is equivocal and suggests that NTG-mediated dilation is either impaired [5, 7, 8] or maintained [2, 6] compared to healthy controls. Our resting NTG responses of 22.0 ± 5.6% are comparable to values reported in healthy populations [5, 29, 38], which suggests that our sample had normal NTG responses during resting conditions. Few studies have examined endothelial-independent function following an acute exercise bout [19, 21, 24, 38], and presently none has demonstrated reduced NTG-mediated dilation following acute exercise. As a result, limited research is available on possible mechanisms explaining the observed responses. A 0.4 mg dose of NTG is believed to elicit the maximum obtainable diameter during resting conditions [4]; however, our data suggests that a maximum obtainable diameter may not have been reached during the resting condition since peak-NTG diameters reached during recovery were significantly larger compared to rest. If there is an upper limit for vasodilation, it was likely reached during the recovery conditions. The attenuation of relative NTG-mediated dilation during recovery could be attributed to the increased pre-NTG brachial artery diameter, and subsequent reduction in the “dilatory capacity” [39]. However, the reduction in NTG-mediated dilation was observed even after “correcting” for pre-NTG diameter, which suggests that there may be additional factors influencing NTG-mediated vasodilation. In particular, based on the observation that the NTG dose given was “submaximal” as the same dose elicited greater dilation postexercise compared to rest, the attenuation of NTG-mediated dilation during recovery may also be attributed to a shift in the dose-response relationship of vascular smooth muscle to NTG after exercise.

NTG administration permits the assessment of the vasodilatory capacity of the vascular smooth muscle; therefore, reductions in NTG-mediated dilation may be attributed to a decreased responsiveness or capacity of the vascular smooth muscle cells to elicit vasodilation. The conversion of NTG to NO occurs in the vascular smooth muscle; therefore, it is unlikely that exogenous NO was scavenged by postexercise increases in superoxide anions. However, there is evidence to suggest that the antioxidant superoxide dismutase, which neutralizes the superoxide anions, is necessary for vasodilation via the activation of soluble guanylate cyclase (sGC) and cyclic guanosine monophosphate (cGMP) [40]. A postexercise increase in superoxide anions beyond the capacity of superoxide dismutase could decrease vascular smooth muscle function by inhibiting sGC and cGMP signaling. Endothelial-independent function may also be affected by postexercise inflammation. Interleukin-1*β* (LI-1*β*) has been shown to decrease endothelial-independent dilation by decreasing sGC activation [41]. Elevated levels of IL-1*β* have been reported following exercise [42]. Further examinations are required to decipher the true mechanism to explain our observation of a postexercise reduction in NTG-mediated dilation.

Time to peak dilation during the FMD and NTG assessments was unaffected by submaximal exercise, suggesting that the time course of brachial artery endothelial responses remained consistent. Peak blood flow and shear rate AUC were also unchanged following acute exercise. Blood flow is regulated by downstream resistance vessels; therefore, our findings suggest that downstream vascular resistance in immediate recovery from submaximal exercise was unaffected. As shear rate AUC is used to quantify the reactive hyperemic stimulus, the absence of a change in AUC suggests a similar hyperemic stimulus across all FMD tests, which would support the validity of our observation of unchanged FMD following acute exercise. The reductions in systolic and mean arterial pressure during recovery are consistent with postexercise hypotension [43] and support the observation of postexercise increases in arterial diameters.

## 5. Limitations

Recovery measurements were performed approximately 45–60 minutes following the first dose of NTG. While there is evidence to demonstrate that repeated FMD tests have no effect on the subsequent test [44], no studies have determined the effect of repeated NTG doses. It is possible the first NTG dose given during resting conditions may have influenced our recovery measurements.

## 6. Conclusions

The combination of endothelial-dependent and endothelial-independent assessments is important because they provide a comprehensive look at the mechanisms involved in peripheral artery vasodilation. NTG assessments are used as an experimental control to measure vascular smooth muscle function. Few previous acute exercise studies have assessed both FMD and NTG responses, which in light of our current findings might limit the ability to assemble an accurate understanding of vascular regulation from previous findings. Even fewer investigations report measurements of both FMD and NTG diameters [17, 24, 32], which may play an important role in determining the dilatory capacity of the artery. We observed a mixture of positive and negative FMD responders during early recovery from submaximal exercise in patients with CAD, which is likely attributed to the degree of resting endothelial dysfunction. NTG-mediated dilation, on the other hand, was significantly attenuated in all patients during recovery. Given that this is the first study to report postexercise reductions in NTG-mediated dilation, further research is required to delineate the mechanisms and clinical significance. Overall, this study identifies a major limitation of previous examinations of acute FMD responses to exercise interventions and highlights the necessity to perform both endothelial-dependent and endothelial-independent assessments for a more global understanding of endothelial function.

## Figures and Tables

**Table 1:** Participant characteristics.

Variable	
Age (yr)	63 ± 8
Height (m)	1.75 ± 0.07
Weight (kg)	87.9 ± 17.0
BMI (kg·m^−2^)	28.6 ± 4.4
Cardiorespiratory assessment	
VO_2_ peak (mL·kg^−1^·min^−1^)	21.2 ± 5.2
Peak heart rate (bpm)	126 ± 21
CAD history (number)	
MI	13
PCI	13
CABG	5
Medication classification (number)	
ACE inhibitors	13
Antiplatelets	19
*β*-blockers	15
Calcium channel blockers	2
Statins	18
Time since CAD event (days)	156 ± 43

Data expressed as mean ± SD. ACE: angiotensin-converting enzyme.

BMI: body mass index.

**Table 2:** FMD, NTG, and hemodynamic indices at rest and recovery.

Variables	Rest	Recovery
FMD indices		
Preocclusion diameter (mm)	4.52 ± 0.76	4.83 ± 0.83*
Postocclusion peak diameter (mm)	4.72 ± 0.75	5.06 ± 0.85*
Time to peak RH diameter (s)	62 ± 29	64 ± 33
Preblood flow (mL·min^−1^)	49 ± 35	56 ± 34
Peak blood flow (mL·min^−1^)	351 ± 177	309 ± 139
AUC (AU)	16586 ± 13081	13855 ± 13817
“Corrected” FMD (%)	4.39 ± 2.20	4.81 ± 2.20
NTG indices		
Pre-NTG diameter (mm)	4.54 ± 0.63	4.93 ± 0.71*
Peak-NTG diameter (mm)	5.52 ± 0.69	5.63 ± 0.72^†^
“Corrected” NTG (%)	20.92 ± 4.46	15.14 ± 4.46*
Hemodynamic indices		
Heart rate (bpm)	57 ± 7	64 ± 8*
Systolic blood pressure (mm Hg)	126 ± 14	118 ± 11^‡^
Diastolic blood pressure (mm Hg)	63 ± 6	62 ± 6
Mean arterial pressure (mm Hg)	84 ± 8	81 ± 6^†^

Data expressed as mean ± SD. AU: arbitrary units. *P < 0.001 versus rest; ^†^ *P* < 0.05 versus rest. ^‡^ *P* < 0.01 versus rest.

RH: reactive hyperemia.

**Table 3:** Comparison of negative and positive FMD responders.

Variable	Negative responders (*n* = 9)	Positive responders (*n* = 10)
Characteristics		
Age (yr)	64 ± 9	61 ± 7
BMI (kg·m^−2^)	28.3 ± 5.0	28.8 ± 4.0
Time since CAD event (days)	162 ± 50	150 ± 37
VO_2_ peak (ml·kg^−1^·min^−1^)	20.4 ± 4.2	21.9 ± 6.1
Submaximal peak heart rate (%HR_max_)	79 ± 18	76 ± 14
Hemodynamic indices		
Rest heart rate (bpm)	59 ± 9	54 ± 4
Rest systolic blood pressure (mm Hg)	129 ± 13	123 ± 14
Rest diastolic blood pressure (mm Hg)	65 ± 6	61 ± 6
Rest mean arterial pressure (mm Hg)	86 ± 7	82 ± 8
Endothelial indices		
Rest FMD (mm)	0.26 ± 0.11	0.14 ± 0.07*
Rest FMD (%)	6.0 ± 2.5	3.2 ± 1.7*
Rest “corrected” FMD (%)	5.9 ± 1.7	3.4 ± 1.8*
Rest NTG (mm)	1.06 ± 0.29	0.91 ± 0.17
Rest NTG (%)	23.8 ± 6.1	20.4 ± 5.0
Rest “corrected” NTG (%)	23.5 ± 4.2	20.4 ± 4.1

Data expressed as mean ± SD. %HR_max_, percentage of age-predicted heart rate maximum. *P < 0.05 versus negative responders.

BMI: body mass index.
